# Complete Genome Sequence of Bacteriophage EO1, Which Infects Both Escherichia coli O157:H7 and *Shigella*

**DOI:** 10.1128/mra.00177-23

**Published:** 2023-05-15

**Authors:** Jieun Choi, Yoonjee Chang

**Affiliations:** a Department of Food and Nutrition, College of Science and Technology, Kookmin University, Seoul, Republic of Korea; DOE Joint Genome Institute

## Abstract

The lytic bacteriophage EO1 has been newly isolated. This phage infects Escherichia coli O157:H7 and has a broad antibacterial spectrum, including against *Shigella*. The complete genome sequence of phage EO1 was determined; its full length is 166,941 bp, and it has a G+C content of 35.46%.

## ANNOUNCEMENT

Escherichia coli O157:H7 is a Gram-negative pathogen that can be a major cause of various clinical diseases, such as hemolytic uremic syndrome, hemorrhagic colitis, and thrombocytopenia (1, 2). Therefore, bacteriophages have been reported as promising biological control agents to target pathogenic bacteria without destroying human, plant, and animal cells (3).

**FIG 1:**
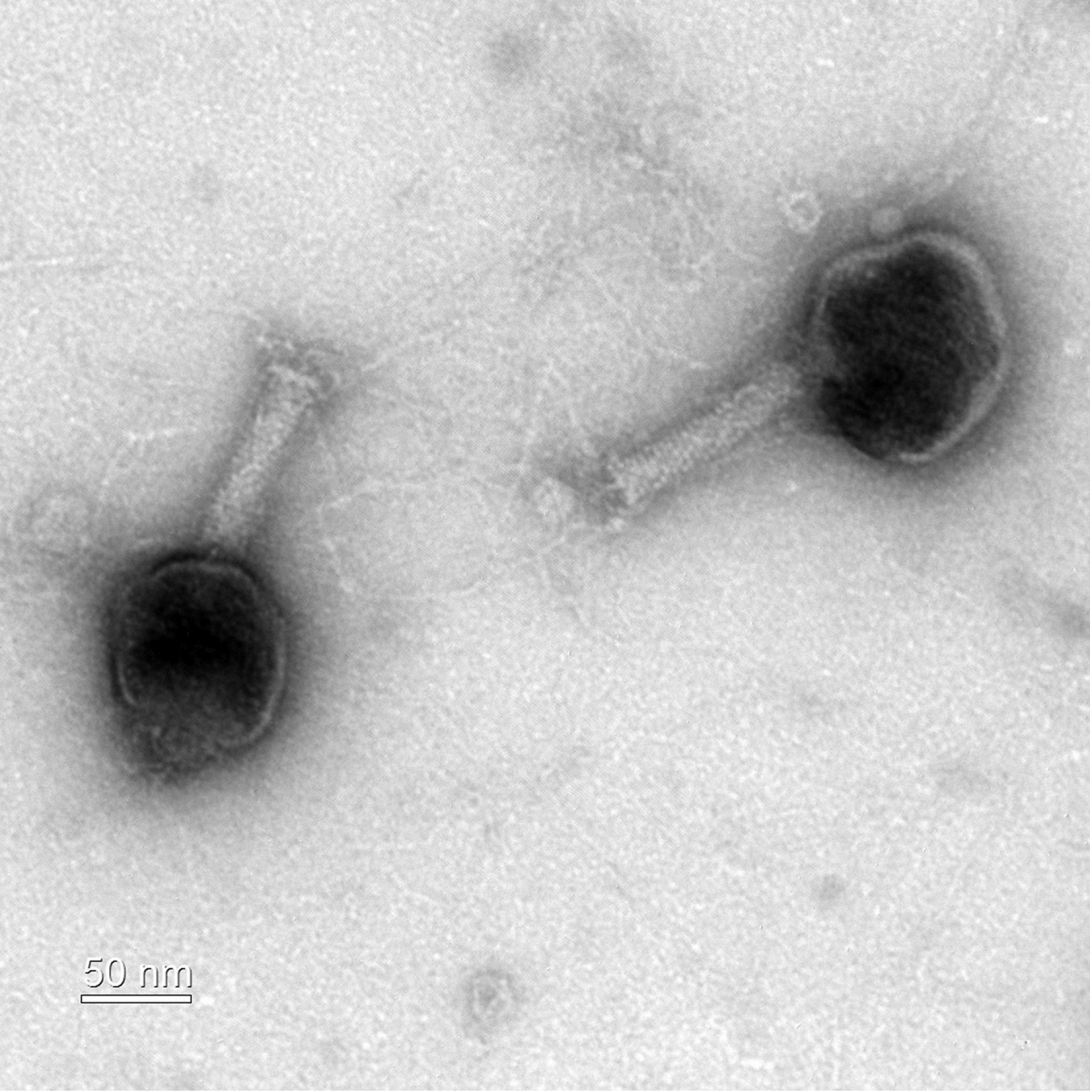
Transmission electron microscopy (TEM) image of E. coli O157:H7 phage EO1. The phage suspension was applied onto a copper grid (200 mesh) and negatively stained with 2% (vol/vol) uranyl acetate (pH 4.5). Phage morphology was observed using an energy-filtering Libra 120 transmission electron microscope (Carl Zeiss, Oberkochen, Germany).

Phage EO1, which forms clear plaques, was isolated in April 2021 from a sewage sample (Tancheon Sewage Treatment Plant, Seoul, South Korea), using E. coli O157:H7 ATCC 43890 as the host bacterium. The sample was grown in Luria-Bertani broth (LB) at 37°C, centrifuged at 15,000 × *g* for 5 min, and filtered through a 0.45-μm-diameter pore syringe filter. Phage single plaques were isolated three times using the spotting assay (4) and double agar overlay method (5). Phage EO1 showed a broad host range, infecting 15 out of 21 different strains tested, including E. coli and *Shigella* spp. (Table 1). Transmission electron microscopy (TEM) images showed the EO1 phage with an icosahedral head of 93 ± 6 nm and a tail of 104 ± 2 nm (*n* = 5) (Fig. 1).

**TABLE 1:** Host range determination of phage EO1^*a*^

Bacterial strain	Sensitivity to lysis by EO1^*b*^	Source or reference
Gram-negative bacteria		
E. coli O157:H7 ATCC 35150	+	KCTC
E. coli O157:H7 ATCC 43888	+	IVI
E. coli O157:H7 ATCC 43890	++	KCTC
E. coli O157:H7 ATCC 43894	++	NCCP
E. coli O157:H7 ATCC 43895	+	KCTC
E. coli ATCC 15597	+	IVI
E. coli ATCC 23724	++	ATCC
E. coli K-12 MG1655	++	ATCC
E. coli DH5α	++	ATCC
Shigella flexneri KCTC 2993	+	ATCC
Shigella. flexneri 2a 2457T	+	ATCC
Shigella flexneri KCTC 2517	+	ATCC
Shigella flexneri NCCP 10852	+	ATCC
Shigella sonnei KCTC 22530	+	6
Shigella boydii IB 2474	+	ATCC
Vibrio parahaemolyticus KCTC 2471	−	KCTC
Salmonella enterica serovar Typhimurium ATCC 14028	−	ATCC
Cronobacter sakazakii ATCC 29544	−	ATCC
Gram-positive bacteria		
Listeria monocytogenes ATCC 15313	−	ATCC
Bacillus cereus ATCC 27348	−	ATCC
Staphylococcus aureus ATCC 29213	−	ATCC

a The spot test (4) was used to determine the host range against 21 strains. Phage lysate (1 × 10^9^ PFU) was spotted onto the bacterial lawns and incubated overnight at 37°C. The efficiency of plating (EOP) was measured as the ratio between the number of plaques on the test bacteria and the number of plaques of E. coli O157:H7 ATCC 43890.

b ++, EOP of 0.1 to 1; +, EOP of less than 0.1; −, no susceptibility to phage EO1.

The SDS-proteinase K method and the phenol-chloroform method were used for phage suspension lysis and DNA purification from lysate after spinning down the cell, respectively (7). A TruSeq Nano DNA library prep kit was used for preparation of the DNA library, and sequencing was carried out on an Illumina MiSeq instrument in 2 × 300-bp paired-end format (8). All tools were run with default parameters unless otherwise specified. Quality trimming was performed using Trimmomatic, and the resulting contigs were assembled by performing *de novo* assembly (SPAdes v.3.13.0). A total of 2,011,220 reads (578,421,935 bp) were trimmed and assembled, resulting in a complete genome sequence with a length of 166,941 bp, a G+C content of 35.46%, and an average coverage of 513.93×. Using RAST (9), GeneMarkS (10), and FGENESV, 139 open reading frames (ORFs) were annotated. The physical ends of the genome were identified by comparing the coverage values of the length of the phage EO1 to closely related phages in the NCBI database, Escherichia phage PEC04 (GenBank accession number KR233165.1; 97.03% identity with 95% query coverage) and Escherichia phage HY01 (NC-027349.1; 97.03% identity with 95% query coverage), using BLAST (11) and ViroBLAST (12).

Based on blastp (13) and InterProScan (14), protein-coding genes predicted to be involved in various functions were classified: structure and packaging (coil, tail fiber protein, tail protein, outer membrane lipoprotein, head chaperone, baseplate hub, major head protein, head scaffolding protein, terminase family protein, neck protein, and large terminase subunit), host lysis (peptidase and holin), DNA metabolism (DNA polymerase, DNA primase, recombinase protein, DNA topoisomerase, DNA ligase, endonuclease, thymidylate synthase, dCTP pyrophosphatase, RNA polymerase, exonuclease, deoxynucleoside monophosphate kinase, and RNA ligase), and additional functions (thioredoxin and naphthalene 1,2-dioxygenase).

Since EO1 forms clear plaques and there are no lysogen-forming genes (e.g., Cl protein or integrase) in the genome, it can be assumed that it is a strictly lytic phage that has great potential for use as a biocontrol agent.

### Data availability.

The complete genome sequence of Escherichia phage EO1 was deposited at GenBank under the accession number OQ223305.1. The associated BioProject, BioSample, and SRA accession numbers are PRJNA942357, SAMN33687158, and SRR24296758, respectively.
